# Comment on “Biomarkers as Potential Treatment Targets in Inflammatory Bowel Disease: A Systematic Review”

**DOI:** 10.1155/2016/3238925

**Published:** 2016-03-31

**Authors:** Nicholas-Paul Delicata, Neville Azzopardi, Pierre Ellul

**Affiliations:** Department of Gastroenterology, Mater Dei Hospital, Msida MSD 2090, Malta

We read with interest the article entitled “Biomarkers as Potential Treatment Targets in Inflammatory Bowel Disease: A Systemic Review,” which brings forward evidence that serum and faecal biomarkers do not possess adequate operational characteristics to make them stand-alone treatment targets in inflammatory bowel disease (IBD) but should be considered as important adjunctive measures to clinical, endoscopic, and radiographic assessment [1].

We recently carried out a study at Mater Dei Hospital, Malta, where we compared the positive predictive value of faecal calprotectin (FC) in patients with newly diagnosed inflammatory bowel disease (IBD), compared to the more widely used erythrocyte sedimentation rate (ESR) and C-reactive protein (CRP). Our patient population consisted of 97 patients with a histological diagnosis of IBD and in whom all three biomarkers (FC, ESR, and CRP) were taken. All faecal calprotectin values were positive (with a mean average of 807 mg/L; normal < 50 mg/L). ESR was only elevated in 45% of cases while CRP was elevated in only 21% of cases. From the 97 patients; 60 were diagnosed with Crohn's disease (CD), 34 with ulcerative colitis (UC), and 3 with indeterminate IBD.

The mean FC level, ESR, and CRP were 857 mg/L, 37 mm/hr, and 30 mg/L in CD; 771 mg/L, 39 mm/hr, and 62 mg/L in UC; 180 mg/L, 40 mm/hr, and 23 mg/L in indeterminate IBD.

There was no statistical significance between the adult and paediatric populations. These results demonstrate and confirm that ESR and CRP are poor markers of intestinal inflammation, when compared to faecal calprotectin. This data suggests that all IBD patients should have a faecal calprotectin measured at outpatients rather than ESR and CRP in order to assess clinical activity even in the absence of gastrointestinal symptoms.

The role of FC in the investigation and management of gastrointestinal pathologies has been on the increase. Nowadays faecal calprotectin is playing a major role in the investigation and diagnosis of patients presenting to the physician with lower gastrointestinal symptoms. When taken appropriately a negative faecal calprotectin would rule out IBD thus sparing most people with irritable bowel syndrome from having costly and invasive investigations [2].

Faecal calprotectin has been demonstrated to correlate better with endoscopic disease activity than clinical activity, CRP, platelets, haemoglobin, and blood leukocytes. Furthermore, the strong correlation of faecal calprotectin with endoscopic disease activity suggested that calprotectin is indeed a useful biomarker for the noninvasive monitoring of disease activity in patients [3]. FC is also useful in predicting the probability of relapses in both quiescent ulcerative colitis and quiescent Crohn's disease [4, 5].

Thus in the context of the ever-increasing financial burden on the health services and the better performance of FC as a biomarker, ESR and CRP should only be requested in exceptional circumstances.
